# A Philosophical Analysis of Non-Markovian Collapse Models

**DOI:** 10.1007/s10838-025-09725-9

**Published:** 2025-11-25

**Authors:** Cristian Mariani

**Affiliations:** Università della Svizzera Italiana, Via Giuseppe Buffi 13, 6900 Lugano, Switzerland

**Keywords:** Non-Markovian dynamics, Quantum mechanics, Spontaneous collapse models, Stochastic noise, Temporal non-locality, Time in quantum mechanics

## Abstract

Spontaneous collapse models (SCM) employ an imaginary noise term in the modification of Schrödinger’s equation in order to achieve a stochastic collapse process. Such noise term is typically assumed to be white, and therefore uncorrelated in time, which results in the dynamics being Markovian. In the first part of this paper I discuss the reasons why physicists are exploring the possibility to instead employ non-white stochastic noise terms in the dynamics of SCM, and I then briefly introduce these models. In the second part, my aim is to provide an evaluation of the major conceptual and philosophical consequences of the generalised collapse models with colored noise. In particular, I will focus on the broad class of implications having to do with the non-Markovian nature of the dynamics, with its resulting temporal non-locality, and on the possibility to consider the noise field as a real physical entity.

## Introduction

Quantum mechanics (QM) has been an extraordinarily successful tool in predicting the behaviour of microscopic phenomena. So successful, that it would be incredibly hard to believe that the theory is not telling us something true about nature. And yet, as we all know, as soon as we start asking very simple questions about what it could be that the theory is telling us, we find no final verdict whatsoever. Disagreement comes at almost every stage, for instance when asking about the ontology of the theory, whether the dynamics is deterministic or not, whether it is time-reversal symmetric, or how we should interpret the non-local correlations that have been experimentally verified. It should not come as a big surprise, then, if you were told that there is yet another, slightly more neglected issue, about which the theory may also be giving us conflicting answers. This concerns another kind of non-locality, one that many find even more disturbing than the spatial one, that is *temporal non-locality*.

In most approaches to QM the dynamics is said to be Markovian. This means that in order to predict future states we just need complete information about the present state. All that happened in the past, and so how exactly we got to that specific present state, does not make a difference for our predictions. For Markovian theories, the past is dead in a very specific way, that is, the past cannot directly influence (in any sense of ‘influence’ you may have in mind that could be dynamically relevant) the future state of the world. This is much more than just an intuitive assumption or a philosophical presupposition. From a purely mathematical perspective, without such crucial Markov property the world would be much harder for us to understand, as the number of variables to take into account while describing a physical system at any given time would increase exponentially, and perhaps uncontrollably. There is a sense in which Markovianity reflects some deeply rooted, hard-wired conception about how we believe things in the world evolve in time and events unfold. Nonetheless, whether or not we live in a world that obeys Markovian laws is still an open issue. As I will show, an ideal place in physics to look at, both in order to understand the importance of Markovianity as well as the costs of its rejection, is the debate on the Spontaneous Collapse Models (SCM) of quantum mechanics.

The SCM are among the major attempts to solve the measurement problem of QM. The key feature of these models lies in the assumption that the wave function undergoes a spontaneous and random process of localisation, and so it collapses without the need to appeal to notions such as those of observer or measurement. To describe how exactly the process of localisation happens to be stochastic, SCM employ an “‘imaginary noise’ trick” (Adler and Bassi 2007b), that is assumed to be white and therefore uncorrelated in time. This means that in all standard versions of SCM, the resulting modified dynamics is Markovian. As already noted, the motivations behind this assumption are of various kind. From a purely mathematical perspective, employing white noise processes with a flat power spectrum allows for much easier mathematical treatments such as the use of differential equations. From a more conceptual perspective, Markovianity appears to be a natural methodological desideratum, since its negation, non-Markovianity, entails a kind of unwanted *– spooky* (Adlam 2018) – temporal action at-a-distance. Despite all of this, in the past couple of decades the possibility to generalize SCM with a non-white noise has been thoroughly explored (Bassi and Ghirardi 2002; Adler and Bassi 2007b; 2008; Diósi 1990; Diósi and Strunz 1997; Pearle 1996; 1999). I will show, in some detail, that there are also several good motivations for considering this possibility, even on the experimental side. As a consequence, at least in the context of SCM, it is fair to say the jury is still out with respect to the issue of whether or not the world we live in is Markovian.

From a broadly naturalistic point of view, I take it that these are reasons enough for philosophers interested in the consequences of QM to seriously entertain the possibility that the dynamics of the world may be fundamentally non-Markovian. As I will show, this possibility has a significant impact on various philosophical views that appear to be fairly standard. Furthermore, by focusing on their non-Markovian versions, we may learn something valuable concerning the ontology of SCM in general. Indeed, as I will show, in the non-Markovian case it is straightforward to treat the noise as representing a real field, as opposed to being just a useful mathematical device that incorporates stochasticity into the equation. Such a realistic interpretation of the noise, however, can naturally be extended to the standard case of white noise SCM, with some interesting consequences.

*Roadmap*. I start in Sect. 2 with a general discussion on the SCM, their main properties, and why they usually employ a white noise. In Sect. 3 I introduce the generalised versions of SCM with a non-white noise, and explain what are the motivations for developing these models. In Sect. 4 I indicate the major conceptual consequences and explanatory challenges of Non-Markovian SCM.

## Noise in Spontaneous Collapse Models

Spontaneous collapse models (SCM; for an overview, see Ghirardi and Bassi 2011) represent one of the major and most discussed attempts to provide a solution to the measurement problem in quantum mechanics. While other approaches leave the Schrödinger equation intact, SCM proposes modifications to the dynamics, and can therefore be considered, strictly speaking, as rival theories to QM. In order to modify the dynamics, SCM adds stochastic and non-linear terms which are meant to explain the collapse of the wave function as a real physical process. The two most prominent SCM are the approaches known as Ghirardi–Rimini–Weber (GRW; Ghirardi et al. 1986) and Continuous Spontaneous Localization (CSL; Pearle 1989). There are several crucial differences between these two, but the most important one concerns the way in which the stochastic collapse occurs as a discrete or as continuous process. In GRW, first proposed in 1986, each particle in a quantum system has a small probability (per unit of time) of undergoing an event of spontaneous localization. These events occur randomly and instantaneously, causing the wave function to collapse to a localized state. The frequency of these events is determined by the collapse rate parameter $\lambda _{GRW}$, typically chosen to be around $10^{-16}$ $\hbox {s}^{-1}$ per nucleon. CSL, developed later, introduces a continuous stochastic process that causes gradual localization. Unlike GRW, which features discrete jumps, CSL describes a smooth and continuous collapse process. CSL also incorporates a mass-proportional coupling, which is designed (among other reasons) to make it more suitable for systems with particles of different types and numbers. In what follows I shall focus on CSL only. This choice is justified in part because of the more developed formalism and wider range of applicability of CSL, and in part because the study of generalised non-white noises, which is the focus of this paper, has been done in that context.^1^

The dynamical equation of CSL can be written as a modified Schrödinger equation of the following form:

1 $$\begin{aligned} \frac{d}{dt}|\psi _t\rangle =\left[ -\frac{i}{\hbar }H+\int d^3x \, w_t(x) M(x)-\frac{\gamma }{m^{2}_0}\int d^{3}x M^2(x)\right] |\psi _t\rangle \end{aligned}$$

The equation describes how the quantum state $|\psi _t\rangle$ evolves over time. The first term in R.H.S. is the standard Schrödinger evolution term, with the Hamiltonian $\hat{H}$ describing the total energy of the system. The third term contributes actively to the collapse mechanism, and also ensures that the normalization of the state is preserved on average. The second term in the R.H.S of Eq. (1), however, is the one that deserves more attention, since it introduces all the crucial properties of CSL. $w_t(x)$ represents the noise field, and I will come back to this in a moment. $\hat{M}(x)$ is the mass density operator, which has been considered crucial by many authors in order to explain what is the ontology of CSL. For some authors, Ghirardi included (Ghirardi and Bassi 2011; Bassi and Ghirardi 2004), we can think of CSL as describing a *mass density field*, which evolves through time in the way given by Eq. (1), meaning it is constantly coupled with a noise field. Finally, $\gamma$ is the collapse rate parameter, and $m_0$ is a reference mass (taken to be that of a nucleon, but which is supposed to vary depending on the particle type). In short, the second term in the dynamical equation of CSL is the one that causes random fluctuations in the quantum state by coupling the noise and mass fields.

Clearly then, the white noise field $w_t(x)$ plays a crucial role in CSL, as it introduces the stochasticity that is necessary to explain the collapse process. Despite its prominent role, philosophers have arguably spent little attention in understanding the ontological meaning of $w_t(x)$, and have rather focused on the *mass density* field. This is surely justified by the thought that the noise field is just meant to be a useful mathematical device that incorporates stochasticity. And indeed, Adler and Bassi (2007b) explicitly call it an “‘imaginary noise’ trick”. The motivation for thinking that the noise field is just ‘a trick’, as opposed to representing something physical, is probably related to the fact that physical noise fields are never white in time. For this reason, philosophers and physicists tend to assume that the noise field is just part of the dynamics (and, in this sense only, it is real) but it is not part of the ontology of the theory.^2^

At this point, I believe it is quite natural to try and better understand what ‘whiteness’ means, both in general and in the context of CSL. To do so, it is helpful to start by considering a simpler case of a white noise process *w*(*t*) that depends on time only. This kind of process would have the following two formal features to define its ‘whiteness’:

1. *Zero mean*. For all times *t*, the expected (or average) value of *w*(*t*) is zero. Mathematically, this is written as:
  2 $$\begin{aligned} \mathbb {E}[w(t)] = 0 \quad \text {for all } t \end{aligned}$$
  Where $\mathbb {E}[\cdot ]$ denotes the expectation value (or average) over many realizations of the noise process. This property basically means that the noise fluctuates around zero, and so that, at any given time, positive and negative values are equally likely.
2. *Delta-correlation*. The correlation between the noise at two different times *t* and *s* is given by the Dirac delta function:
  3 $$\begin{aligned} \mathbb {E}[w(t)w(s)] = \delta (t-s) \end{aligned}$$
  This requires some unpacking. The Dirac delta function $\delta (t-s)$ is a generalized function that is zero everywhere except at $t=s$, where it is infinitely large, and its integral over any interval containing the point $t=s$ is equal to 1.
  What eq. (3) tells us then, is that the noise values at any two different times are completely uncorrelated. If $t \ne s$, then $\mathbb {E}[w(t)w(s)] = 0$, and therefore knowing the value of *w*(*t*) gives us no information whatsoever about *w*(*s*). If instead we take $t=s$, the noise is perfectly correlated with itself in a sense captured by the properties of the Dirac delta function.

Already at the stage of such a simple case, there are several important implications to consider and to keep in mind for what follows. First, such a noise will fluctuate in a incredibly rapid way. And in fact, a true white noise process is not a function in the usual sense; take any point, and it will not be measurable at that point. It is exactly for this reason that it should be thought of more as a mathematical idealization, useful for modeling purposes (a ‘trick’, to use Adler and Bassi’s expression), than as a physical and measurable field. Second, white noise has equal power at all frequencies (which is also why it is called *white*, in analogy with the power spectrum of white light). The meaning of this, from a physical point of view, is that a noise that is white will excite or perturb a system interacting with it at all frequencies equally. Third, because of *Delta-correlation*, a white noise process has no *memory* of previous interactions or states. Its value, at any instant, is completely independent from, and uncorrelated to, its values at any other given time. Each of these three key properties of white noise processes plays a significant role. From now on, I will call them, respectively (i) *non-physicality*, (ii) *equality*, and (iii) *memorylessness*.^3^

Let us now consider the type of noise we are dealing with in the context of CSL. While in the previous, simplified case we were only considering a noise that depends on time *t*, here we consider a field $w_t(x)$ that also depends on position *x*. Still, the properties of this field appear to be a natural extension of the time-only case^4^:

4 $$\begin{aligned} \mathbb {E}[w_t(x)]= & 0 \quad \text {for all } t \text { and } x \end{aligned}$$

5 $$\begin{aligned} \mathbb {E}[w_t(x)w_s(y)]= & \delta (t-s)\delta (x-y) \end{aligned}$$

The second equation, (Eq. 5), now involves delta functions in both time and space.^5^ This means that the noise is uncorrelated not only across different times but also across different spatial points. In other words, knowing the value of the noise at any given point in space or time tells us nothing about its value at any other point in space or time. This spatiotemporal white noise can be visualized as a field that fluctuates wildly and randomly at every point in space, with these fluctuations being completely independent from point to point and from time to time. Such a feature is crucial for CSL, for it allows the model to induce collapses of the state that are *local* at least in the sense that they affect different parts of the wave function independently.^6^

The three key properties of the white noise field individuated above extend naturally to the case of CSL. As for *non-physicality* and *equality*, it is important to note that while white noise is mathematically convenient and leads to a Markovian dynamics, it is an idealization that cannot be thought to be realized in actual physical systems. Real noise always has some correlation in time, which is one of the motivations for considering non-white (colored) noise in more advanced collapse models. As for the property I called *memorylessness*, the use of white noise in CSL is intimately connected to the Markovian nature of the resulting dynamics. To understand this connection, we need to explore what Markovian dynamics means and how white noise leads to such dynamics.

A process is called Markovian if its future evolution depends only on its current state, not on its past history. In the context of quantum mechanics, for a system described by a density matrix $\rho (t)$, Markovian evolution can be mathematically expressed as:

6 $$\begin{aligned} \rho (t_2) = \mathcal {T}(t_2, t_1) \rho (t_1) \end{aligned}$$

Here, $\mathcal {T}(t_2, t_1)$ is a map that takes the state at time $t_1$ to the state at a later time $t_2$. Crucially, this map depends only on the time interval $(t_2 - t_1)$, not on the absolute times or any earlier history of the system. The key property of white noise that leads to Markovian dynamics is its delta-correlation in time (Eq. 5). This property means that the noise at any given time is completely uncorrelated with the noise at any other time. In other words, the noise has no “memory” – its value at one instant tells us nothing about its value at any other instant.

When we use such noise in the CSL equation, it means that the random influence on the system’s evolution at any moment is completely independent of the random influences at all other moments. This lack of temporal correlation in the noise translates into a *lack of memory* in the system’s dynamics. As a result, the evolution of the quantum state from one moment to the next depends only on the current state and the current value of the noise, not on the system’s history or in the values of the noise in the past. This is in a nutshell what defines a Markovian dynamics.

This Markovian property allows us to describe the evolution of the system using differential equations that are local in time. For CSL, we can derive a master equation for the density matrix that takes this form:

7 $$\begin{aligned} \frac{d\rho }{dt} = -\frac{i}{\hbar }[H, \rho ] - \frac{\gamma }{m_0^2}\int d^3x \, [\hat{M}(x), [\hat{M}(x), \rho ]] \end{aligned}$$

This equation describes how the density matrix $\rho$ changes in an infinitesimal time step, and it depends only on the current state $\rho$, not on the system’s history. And this is, again, the hallmark of Markovian dynamics.

The Markovian nature of white noise CSL models greatly simplifies their mathematical treatment, for it allows us to use powerful and well-known computational techniques such as the theory of Markov processes. However, it is important to stress that the Markovian property, while mathematically convenient, is an idealization. I take this to be particularly important as soon as we begin asking questions about the ontology posited by CSL. Very often, philosophers tend to disregard the importance of the noise field as a physical entity, and rather focus on the mass density only. This happens despite the fact that, if we look at face value at the dynamical equations, the two fields, by being coupled, are treated equally. However, physical noise fields typically have some degree of temporal correlation which lead to non-Markovian effects. And this is the core motivation for considering colored noise in more advanced collapse models, as we are about to explore in the next section.

## Generalization to Non-White Noise

Despite the mathematical convenience and widespread use of white noise in SCM, these models face several significant challenges. These arise from the idealized nature of white noise and its implications for the physical interpretation of the models, and can be classified into four distinct kinds of issues.

First of all, as stressed by several authors (see Ghirardi 2016, for a review), white noise SCM predict an unbounded increase in the energy of physical systems over time. Although incredibly small, this energy increase appears to be physically problematic for several reasons. First, it violates the principle of energy conservation, a cornerstone of physics. Second, it predicts that all matter should be constantly heating up due to the collapse process, which is not what we observe in nature. Third, it leads to potential difficulties in formulating a relativistic version of the theory, as infinite energy is incompatible with special relativity.

Second, and relatedly, the instantaneous correlations of white noise in space could potentially allow for superluminal signaling, conflicting, once again, with the principles of relativity. This issue becomes particularly problematic when attempting to construct relativistic versions of SCM (Bassi and Ghirardi 2004).

Third, there are significant issues with white noise models having to do with experimental constraints. Some proposed collapse rates, such as those suggested by Adler (based on latent image formation in photography), would lead to observable effects in white noise models that have not been seen experimentally. For instance, Adler (2007a) proposed a collapse rate about $10^9$ times larger than the original GRW value. With white noise, such a large collapse rate would lead to noticeable heating effects and other anomalies in various physical systems. The fact that these effects have not been observed puts strain on white noise SCM, suggesting that either the collapse rate must be much lower (potentially making the models less effective at solving the measurement problem) or that the noise model needs to be modified.

Fourth, as I already indicated, white noise is a mathematical idealization, and, as such, it is unclear whether we should think of it as something realized in actual physical systems. Stochastic processes always have some correlation in time, no matter how short. This means that white noise SCM, while mathematically convenient, are using a fundamentally un-physical noise source. This raises questions about the physical interpretation of these models, or at least puts seriously into question their status as fundamental theories.^7^

These challenges highlight the tension between the mathematical simplicity of the white noise models and their physical plausibility, thus motivating the exploration of more sophisticated models with colored noises. Following Bassi and Ghirardi (2002), and Adler and Bassi (2007b), we could formulate a generalized stochastic Schrödinger equation for non-Markovian CSL which takes the following form:

8 $$\begin{aligned} \frac{d}{dt}|\psi _t\rangle =\left[ -\frac{i}{\hbar }H+\int d^3x \, w_t(x) M(x) dt-\frac{\gamma }{m_0^2} \int d^3xy M(x)g(|x-y|)\int _{t_0}^t h(t-s) \frac{\delta }{\delta w(y,s)} ds \right] |\psi _t\rangle \end{aligned}$$

The first two terms of the R.H.S. in Eq. (8) are the same we had in Eq. (1), and so the crucial differences are in the third and last term. While *w*(*x*) still represents the stochastic field, the way it interacts with the mass *M*(*x*) is now mediated by two novel correlation functions, a spatial one and a temporal one, given respectively by $g(|x-y|)$ and $h(t-s)$.^8^

The key idea in generalizing CSL is to replace the white noise field $w_t(x)$ with a colored noise field that has non-trivial temporal and spatial correlations. This colored noise field, denoted as $w_c(x, t)$, satisfies:

9 $$\begin{aligned} \mathbb {E}[w_c(x, t)]= & 0 \end{aligned}$$

10 $$\begin{aligned} \mathbb {E}[w_c(x, t)w_c(y, s)]= & D(x - y, t - s) \end{aligned}$$

where *D*(*x*, *t*) is a correlation function with non-zero extent in space and time, and it is defined as $D(x, t) = g(x)f(t)$, where *g*(*x*) is the same spatial correlation function (as in standard CSL), and *f*(*t*) is a temporal correlation function.^9^ Technical details aside, the choices for *f*(*t*) are such that the temporal correlation may vary, for instance exponentially, or in a smooth way describing a Gaussian around zero (Bassi and Ghirardi 2002).

Equation (8) is an integro-differential equation because it involves both integrals (over space and past time) and derivatives (including functional derivatives). This structure encapsulates the very essence of the non-Markovian dynamics. The third term of Eq. (8), just described, is the one that allows for memory effects in the dynamical evolution. The integral over time from 0 to *t* basically means that the entire history of the system contributes to its current state and future evolution. The functional derivative $\frac{\delta }{\delta w_c(y, s)}$ represents how the state vector responds to changes in what the state of the noise field was at earlier times. Therefore, contrary to what happens in standard CSL, where the noise $w_t(x)$ is coupled with the mass *M*(*x*) at each time indifferently from what happened earlier, in the generalised models the mass field is affected by the state of the noise in the past.

These generalised non-Markovian collapse models exhibits several important features that distinguish them from standard CSL. To better appreciate the differences, recall from above the three main properties of white noise CSL which I named (i) *non-physicality*, (ii) *equality*, and (iii) *memorylessness*. Starting with (i), the colored noise field of the generalised models does not appear to be simply an idealization anymore, and it may be interpreted as a real physical field which interacts with the mass density. I will come back to this later on in Sect. 4.2. As for the property (ii) of *equality*, the rate at which superposition states collapse in the non-Markovian models depends on the spectral properties of the noise, and so it is interestingly different from the standard versions. This allows for much more flexibility in matching theoretical predictions with experimental constraints, and opens up new possible venues for experimental tests.^10^ Finally, recall that in white noise CSL the system has no *memory* of previous interactions or states (property (iii), which I called *memorylessness*), for the values of the noise field, at any instant, is completely independent from its values at any other given time. On the other hand, non-Markovian dynamics introduce memory effects, in the sense the future evolution of the system depends on its past history and on the values of the noise field on previous times.

## Conceptual and Philosophical Consequences

I have shown that there are several good motivations, even on the experimental side, for developing collapse models with generalised non-white noise. As a consequence of this, I think that any philosopher who takes seriously SCM as a viable solution to the measurement problem should start considering what are the major conceptual consequences of these generalised versions. These extend well beyond the technical aspects of the models, and touch on fundamental metaphysical questions.

In what follows I decided to restrict my attention only on two among the major philosophical consequences of these models. The first issue I am about to discuss, in Sect. 4.1, has to do with the broad class of ontological and metaphysical implications stemming from Non-Markovianity. The emergence of memory effects in the dynamics poses a series of conceptual puzzles, and puts into question our standard understanding of several familiar notions such as causality, randomness, and temporal extension. The second issue, which I will discuss in Sect. 4.2, has to do with the ontological interpretation of the noise field. As I have shown, while in the context of white noise models the noise field is generally interpreted as a useful mathematical device, in the non-white cases it appears to be possibly related to some real physical field. However, as soon as we realise that the kind of interaction between noise and mass fields is identical in both cases, we can begin reinterpreting the nature of the noise field in all SCM in general, independently from the color of the noise.

Of course, I am well aware that restricting my attention only to these two topics will leave some other important issue untouched. In particular, I will deliberately avoid to comment on the analogies and differences between non-Markovian SCM and other non-Markovian approaches to be found in the debate on quantum foundations.^11^ Similarly, I will not discuss in depth the possible conceptual relationships between temporal and spatial non-locality,^12^ and the possible impact of non-Markovianity on the theory of measurement^13^, and on debates on retro-causality.^14^ I leave the discussion of these and other important issues to future works.

### The Consequences of Non-Markovianity

Probably the most striking feature of the non-Markovian collapse models is their inherent *temporal non-locality*. The evolution of a physical system, at any given time, depends not just on its current state, but on its entire history up to that point. This feature appears to have a significant impact on our pre-theoretical understanding of certain time-related notions.

The first, perhaps natural consequence one could draw, is to connect the non-Markovianity to the debate on temporal ontology. From this point of view, it may be tempting to argue that non-Markovian models, assuming that they provide a true description of nature, entail that the past is *as real as* the present. For instance, Adlam (2018) argues that non-Markovian models entail a rejection of presentism – according to which only the present exists. If the past no longer exists – so the thought goes – it surely cannot actively influence what happens. Conversely, but on a similar spirit, Builes and Impagnatiello (forthcoming) recently provided an argument for presentism based on the assumption that our best physical theories are Markovian. Both lines of reasoning just mentioned, while pointing at different directions, appear to share the conviction that Markovianity or its negation have an impact on what exists in the temporal sense. This conviction, in turn, is based on the more general assumption according to which properties of the dynamics can teach us something about ontology. Similarly to what happens in the context of the debate on the ontological consequences determinism and indeterminism (see, e.g., Pooley 2013), however, this assumption can be challenged in many ways.^15^ In general, whether or not we are allowed to use certain specific feature of the laws in order to argue for an ontological position, turns out to depend on what we think laws of nature are in the first place. Details aside, and to keep things as simple as possible, there is at least one big divide which matters here. For those (like Humeans) who believe that laws are nothing over and above particular facts, and that they are the most informative and simple way to systematize regularities between those facts, the properties of those laws are likely to have no impact whatsoever on what exists. By saying, for instance, that our laws of physics are non-Markovian, we are simply acknowledging the fact that the information required to predict future states based on the present state is, in some sense, incomplete. Conversely, if one believes (along with most anti-Humeans) that laws of nature have the active role of *determining* (in a sense that needs to be specified further) what exists, then of course the properties of those laws (the precise ways in which the *determination* occurs) have an impact on our ontological views.

Given all this, it is fair to say that the arguments leading to a rejection of presentism based on non-Markovianity are not as straightforward as we may have thought. At best, they depend considerably on other independent assumptions regarding the nature of laws. But there is yet another way for presentists to escape this line of reasoning. Indeed, I believe it is crucial that we preliminarily agree on what it is that presentists take their view to be essentially about. If we consider presentism (Ingram and Tallant 2022) as a view about what exists (rather than as a view about what is present), we could argue that non-Markovianity simply forces us to extend the content of the present. This may suggest a kind of “extended present” which continues to influence the future in a direct way, and beyond merely setting initial conditions. Though not necessarily opposed to the letter of presentism, however, I take it that many would agree that the resulting view betrays its spirit. In conclusion, although there is not certainly a knock-down objection to presentism from non-Markovianity, it is certainly true that presentism faces some explanatory challenge that needs to be addressed, whereas forms of eternalism do not face such challenges at all. However, regarding eternalism, we should keep in mind that non-Markovian SCM are inherently *indeterministic* models. Therefore, any alleged compatibility with *eternalism* would presuppose a certain understanding of laws of nature such that, on that understanding, the ontology of the block is compatible with the indeterministic nature of the dynamics. So, once again, not all views on laws of nature would be compatible. On a non-Markovian and indeterministic eternalist models, the past would not be entirely fixed and given once and for all, but would rather continue to contribute actively to the present and future.^16^

The introduction of colored noise and non-Markovian dynamics also has significant implications for our understanding of causality and counterfactual dependence. In Markovian theories, causality is rather straightforward: future events are caused by events that lie in their past. Non-Markovian models seem to complicate this picture. While they do not allow for backward causation (the future influencing the past), they do allow for the possibility that past events continue to influence present and future ones. As a result, it is an incredibly hard task that of providing an efficient semantics for counterfactual claims, since this is usually based on fixing the initial conditions, so to say, directly in the antecedent.^17^ If the entire history of a system is possibly influencing its future evolution, it is unclear how we are supposed to fix the antecedent of a counterfactual in any reasonable way. This may require some sort of revision of our accounts of causation and counterfactuals to accommodate for such complex cross-temporal dependencies. All else being equal, of course, requiring such a revision is a huge limitation of the non-Markovian models.

### The Reality of the Noise Field

The main conceptual puzzles that arise from considering non-white CSL models seriously from an ontological perspective surely have to do with the non-Markovian nature of their dynamics. Nonetheless, there is another profound implication that has gone quite unnoticed. This has to do with a view I shall call *noise-field realism*, and with the possibility of considering the noise field as representing a physical entity.

In standard collapse models – on this regard, CSL and GRW can be treated together – the noise field is considered just a useful mathematical device that incorporates stochasticity in the dynamical equation. However, if we look *at face value *at what the dynamics says, such noise field is coupled with the mass density field (which is usually considered to be the *ontology* of the theory^18^), and so it *interacts* with it. Therefore, one would be tempted to suggest that the noise is actually representing, directly, some physical field, and that it is therefore not simply part of the dynamics.^19^ As we saw already, however, such position is not generally defended in the literature. Perhaps, the reason is once again that in standard CSL the noise is white, which makes it look like as some kind of artificial device that we use in order to achieve the stochasticity. But even granting that such anti-realistic attitude is justified in the white noise case, it surely ceases to be justified once we look at the non-Markovian models introduced in Sect. 3. In these models the noise field looks like a real physical field, with a given power spectrum and precise spatial and temporal correlations with the mass density. Changing the field has a significant impact on the ontology, in ways that we can in principle test experimentally. A similar double standard in the interpretation of the noise field comes up quite clearly in the following two passages from Adler and Bassi (2008):

[...] a non-white noise, unlike a Wiener process, can be identified with a physical field; accordingly, one can try to connect the collapse mechanism to some other physical process occurring in Nature. (3)

the white noise formalism is robust under a generalization to the physically more realistic assumption of non-white noise. (17)

In these passages it clearly emerges the contrast between the attitudes towards white and non-white noise fields, with the latter only being given a physically realistic interpretation. However, as it should be clear by now, any reason one could have to possibly interpret the noise field realistically in the non-white case, is also equally present in the white noise case. After all, the kind of interaction that we see in the dynamics is identical in the two cases. Therefore, provided that we have reasons to believe that the field is real in the non-white case, we thereby also have reasons to believe in its reality also in the standard, white noise models.

By interpreting the noise field (be it white or colored) as part of the ontology, rather than simply as a dynamical feature, we end up with an interestingly novel view, which I tentatively call *noise-field realism*. On this view, the role of the wavefunction is to give information about how the two physical fields (the *mass* and the *noise*) interact with one another. This is an interesting conclusion in itself, since it could provide a different understanding of the nature of the wave function in SCM, and in effect, there has been a quite long debate on this issue (see, e.g., Lewis 2006). To the best of my knowledge, in these debates the nature of the noise field has not been much discussed. Usually, this debate revolves around a quite common and general divide – and so, independent from SCM in particular, but having to do with any approach to QM – on the meaning of the wave function as possibly having some nomological role (see Chen 2019 for an overview). On some views, the nature of the noise field appears to be related to the dynamics, rather than the ontology (see Allori et al. 2008). As a result, if, on these views, the ontology only consists in the mass density field, it appears to be quite hard to explain how the wave function could have an active role in determining physical phenomena. An alternative view is to accept that the role of the wave function, rather than being nomological, is to functionally tell us how two physical fields (the noise and mass density) interact with one another. The wave function is nothing but that the thing which tells us how the two fields interact, and it does not by itself have any physical or nomological role whatsoever.

Of course, this view also comes at a cost, because the noise field is given an ontological interpretation which is much less obvious than that of the mass density field. There may be several ways in which, on a view like this, we may attempt to understand the nature of the noise field. In the generalised colored noise CSL, one would be tempted to believe that whatever the noise source is, it must be something physical. As Adler and Bassi (2008) hints at, the realistic interpretation of the noise field could perhaps be linked to some other, so far not fully understood physical process (gravitational, for instance). In this case the noise field would be part of the derivative ontology, as it would emerge from some underlying, partially unknown, physical process. That is not however the only position available, as we could instead believe that the noise just is a fundamental entity (or, at least, *as fundamental as* the mass density is). Anyways, as of now, and without knowledge of what the noise field could be representing, it is certainly a mysterious posit in the ontology. What is certainly an interesting consequence of this whole analysis is that the three viable options (*dynamical*, *ontological and derivative*, or *ontological and fundamental*) for interpreting the noise field, are equally available in the case of standard white noise models and in the colored ones. All of this, I believe, suggests that the *noise-field realism* view should be considered an option to analyse and to develop further.

## Conclusions

In this paper I have explored the philosophical implications of non-Markovian spontaneous collapse models (SCM) of quantum mechanics. I began by introducing standard SCM, which employ white noise to induce wave function collapse, and I discussed the key property of that noise: *non-physicality*, *equality*, and *memorylessness*. I then examined the motivations for generalizing these models to incorporate colored noise. In the final part of the paper, I focused on two major philosophical consequences of non-Markovian SCM. First, I analyzed the ontological and metaphysical implications of non-Markovianity, which introduces memory effects and temporal non-locality into the dynamics of quantum systems. As I have shown, this *prima facie* challenges traditional notions of causality and counterfactuals, and could potentially provide arguments for rejecting views in temporal ontology. Second, I introduced the view of *noise-field realism*, which emerges from the consideration of colored noise as a physical entity rather than a mere mathematical device. This new perspective opens up new possibilities for interpreting the ontology of SCM and the functional role of the wave function.

## Footnotes

1 Though see Ghirardi (2016) for a brief discussion of white noise in GRW.

2 I will say more on this in Sect. 4.2.

3 White noise processes are ubiquitous in physics and other fields. Some examples include the thermal noise in electrical circuits, the shot noise in electronic devices, or the idealized force acting on a Brownian particle. The Wiener process, also known as Brownian motion, is closely related to our standard understanding of the white noise. It can be thought of as the integral of a white noise process, $W(t) = \int _0^t w(s) ds$, with the following properties: (i) $W(0) = 0$ almost surely; (ii) for $0 \le s < t$, the increment $W(t) - W(s)$ is normally distributed with mean 0 and variance $t-s$; (iii) the increments $W(t_2) - W(t_1)$ and $W(t_4) - W(t_3)$ are independent for non-overlapping intervals $[t_1, t_2]$ and $[t_3, t_4]$.

4 What follows is simplified case. Note however that, in the context of SCM, we would also need to specify a Gaussian distribution to characterize the noise ($\mathbb {E}[w_i(t)] = 0, \quad \mathbb {E}[w_i(t)w_j(s)] = \gamma \delta _{ij} \delta (t-s)$). Strictly speaking, however, this property is independent from the color of the noise.

5 I am again keeping things as simple and schematic as possible here. However, to be precise, in CSL we would need to specify the constant $\frac{\gamma }{m^{2}_0}$ in the R.H.S. of Eq. (5).

6 Despite the fact that the noise itself is local in the sense just described, collapse in CSL is not, strictly speaking, fully local, because the Gaussian mediating the coupling of the noise and of *M*(*x*) has a certain width.

7 This may also be related, I believe, to the worry that the SCM are *ad hoc* Also notice that, for these reasons, one could instead insist that the models are merely effective approximations of some underlying reality (which is supposed to also explain the nature of the noise).

8 Bassi and Ghirardi (2002) provide details on how to provide specific forms of these correlations function.

9 As above, I once again deliberately omit the constant $\frac{\gamma }{m_0^2}$ for simplicity.

10 Possible venues of experimental testings discriminating Markovian and non-Markovian models may have to do with matter-wave interferometry, optomechanical systems, spontaneous X-ray emission, and cold atoms experiments. In all of these, the key challenge is to distinguish the physical effects of collapse from effects coming from other sources. See Bassi and Ghirardi (2004, Part V) for a general introduction, and Toroš et al. (2017) for a recent review.

11 For instance, the theory of open quantum systems (see Breuer and Petruccione 2007), which describes the interaction of a quantum system with its environment, often involves non-Markovian dynamics. The key difference is that in open systems theory, the non-Markovianity arises from the interaction with a complex environment, while in collapse models it is a fundamental feature of the dynamics.

12 See Adlam (2018) for a discussion on this. Just as entanglement allows for non-local correlations in space, the memory effects in non-Markovian models may allow for non-local correlations in time. Recent work on quantum temporal correlations, including the development of so-called “causal Bell inequalities” (Branciard et al. 2015) has highlighted the importance of temporally non-local effects in quantum mechanics. Non-Markovian collapse models may provide a concrete physical mechanism and an explanation for generating such correlations.

13 In the standard understanding, measurement is often treated as an instantaneous process that causes immediate collapse of the state. Non-Markovian models suggest instead a more gradual, history-dependent process of collapse, blurring the line between measurement and ordinary dynamical evolution. This clearly has profound implications for our understanding of the measurement process in quantum mechanics. If collapse really is gradual, it is unclear whether we can always speak of definite outcomes of given measurements (until, so to say, the collapse has reached completion). It appears that the properties of physical systems themselves, on this understanding, can somehow be treated as property of the systems’ whole history, rather than as property of the system at some given time. The differences between Markovian and non-Markovian models in this respect deserves further investigation, especially since we should keep in mind that in the context of SCM the line between measurements and dynamical evolution is already quite blurred. I thank a reviewer here.

14 See again Adlam (2018) for some discussion on this. Some approaches to quantum foundations, such as the two-state vector formalism or the transactional interpretation, incorporate a kind of “retrocausality” where future events can influence the past. While non-Markovian collapse models do not involve true retrocausality, their incorporation of memory effects and extended temporal correlations bears some conceptual similarities to these approaches.

15 I am deeply thankful to a reviewer of this journal for inviting me to say more on this issue.

16 This is perhaps not totally unprecedented, as it has interesting parallels with some interpretations of quantum mechanics, such as the transactional interpretation (Cramer 1986), which posits a *handshake* between past and future events. The kind of *indeterminacy* in the past is however quite novel, and does not resemble to any form of objective indeterminacy already studied in the literature (see, e.g., Calosi and Mariani 2021).

17 Though, to be clear, at least on Lewis (1973) proposal, non-Markovianity is not excluded.

18 Assuming, as most people do, that the ontology of CSL is at least in part given by the mass density function. For instance, Ghirardi and Bassi (2011) do so explicitly.

19 Here I am assuming a certain way of understanding theories, which roughly follows Maudlin (2018), according to which a clear presentation of a physical theory consists in specifying (i) first, the ontology which should be explained in non-dynamical terms, and then, (ii) only afterwards, the given dynamics. On this approach, it follows that the noise field is either part of the ontology or part of the dynamics. Of course though, by being part of the dynamics, the noise field is no way *less real* I thank an anonymous referee of this journal for inviting me to be clear on this.
